# Metallothionein ameliorates burn sepsis partly via activation of Akt signaling pathway in mice: a randomized animal study

**DOI:** 10.1186/s13017-015-0044-3

**Published:** 2015-11-05

**Authors:** Keqin Luo, Huibao Long, Bincan Xu, Yanling Luo

**Affiliations:** Department of Emergency, SunYat-Sen memorial Hospital, Sun Yat-Sen University, 107 yan-jiangxi Road, Guangzhou, 510120 China

**Keywords:** Metallothioneins, Burn, Sepsis, Inflammation, Akt

## Abstract

**Introduction:**

Metallothioneins (MTs) are a family of cysteine-rich and low molecular-weight proteins that can regulate metal metabolism and act as antioxidants. Recent studies showed that MTs played a protective role in excessive inflammation and sepsis. However, the role of MTs in burn sepsis remains unclear. This study is designed to investigate the role of MTs in burn sepsis in an experimental mouse model.

**Methods:**

MT-I/II knockout (−/−) mice on a C57BL/6 background and their wild-type (WT) littermates were randomly divided into sham burn, burn, burn sepsis, Zn treated and Zn-MT-2 treated groups. Levels of inflammatory cytokines were measured by enzyme-linked immunosorbent assay (ELISA). Myeloperoxidase (MPO) activity was detected by spectrophotometry. In in vitro study, exogenous MT was added to macrophages that stimulated with serum from burn sepsis mice with or without Akt inhibitor LY294002. The IL-1 β and IL-6 mRNA expression were detected by quantitative real-time polymerase chain reaction. The levels of Akt expression were determined by western blot.

**Results:**

Burn sepsis induced significantly elevated levels of inflammatory cytokines in serum and increased inflammatory infiltration in the liver and lung. These effects were more prominent in MT (−/−) mice than in WT mice. Furthermore, exogenous MT-2 inhibited these elevated inflammatory response in both WT and MT (−/−) mice. MT-2 up-regulated Akt phosphorylation and abrogated the increase of IL-1β and IL-6 mRNA expression from macrophages that stimulated with burn sepsis serum. These effects of MT-2 were abolished in the presence of LY294002.

**Conclusion:**

MT-2 ameliorates burn sepsis by attenuating inflammatory response and diminishing inflammatory organ damage, which is at least partly mediated by activation of Akt signaling pathway.

## Introduction

Sepsis remains the leading cause of death in patients who have suffered a severe burn injury, despite advances in antimicrobial therapies have been made [1]. The excessive proinflammatory response after burn is reported to be an important driving factor to the pathogenesis of sepsis [2]. Burn injury initially evokes a pro-inflammatory cascade, if uncontrolled, then subsequent development of systemic inflammatory response syndrome, susceptibility to sepsis, and multiple organ damage would occur, which will largely determine the morbidity and mortality of major burn injuries [3].

Metallothioneins (MTs), characterized by very small molecular weight, high cysteine content, and high affinity to divalent metals, were discovered from horse kidney about five decades ago [4, 5]. In mice, there are four main isoforms of MT: MT-I to MT-IV [6]. MT-I and MT-II are almost expressed in most organs, MT-III is mainly expressed in the brain and reproductive systems, and MT-IV is mainly seen in stratified squamous epithelial cells [7–9]. In humans, there are 10 functional isoforms of MTs, which can be subdivided into four groups: MT-I, MT-II, MT-III and MT-IV [10].

MTs have been known to exert diverse effects, such as regulating intracellular metal metabolism, protecting cells against oxidative damage, and serving as a reservoir of heavy metals [11]. Recently, MTs are reported to play important roles in many inflammatory conditions or diseases. For example, MTs inhibited the expression of proinflammatory proteins and protected against LPS induced lung injury and multiple organ damage [12]. Moreover, MTs are reported to play beneficial roles in brain inflammation [13], cardiac dysfunction in sepsis [14], and experimental colitis [15]. However, to date, the role of MTs in burn sepsis remains unclear.

In the present study, we hypothesized that MT may play a beneficial role against burn sepsis by inhibiting excessive inflammatory response and attenuating inflammatory organ damage. So we investigated the role of MT in burn sepsis using an experimental mouse model. Specifically, we chose a most commonly used subtype of MTs, Zn-MT-2 for the treatment intervention. Furthermore, we used Zn alone as a control because Zn was reported to display protective effects independent of MT-2 [16]. At last, we discussed whether the effect of MT was mediated by the Akt signaling using an Akt inhibitor 2-(4-Morpholinyl)-8-phenyl-4H-1-benzopyran-4-one hydrochloride (LY294002).

## Methods and materials

### Animals

MT-I/II double knockout (−/−) mice on a C57BL/6 background and their wild type (WT) littermates (male, 20 ± 1 g, 6–7 weeks old) were purchased from Model Animal Research Center of Nanjing University, Jiangsu, China. Mice were housed conventionally in a constant temperature (25 °C) and humidity (50–60 %), under a 12-h light/dark cycle. Mice were given free access to food and water, and were housed for 7 days before the experiments were started. The research protocol was approved by the Committee of Scientific Research of SunYat-Sen memorial Hospital, Guangzhou, China.

### Experimental burn model

WT mice and MT-I/II (−/−) mice were randomly divided into the following groups: sham burn, burn, burn sepsis, Zn treated and Zn-MT-2 treated groups (10 mice in each group). Mice were anesthetized by intraperitoneal injection with 50 mg/kg pentobarbital sodium. The hairs of the dorsal area were removed. To create a full-thickness burn injury, mice were placed in a template immersing 25 % of the total body surface area (TBSA) in 100 °C water for 10 s [17]. Mice were then resuscitated by intraperitoneal injection with 40 mL/kg normal saline and placed in individual cages with free access to food and water. Mice in sham burn group were treated in the same manner except that they were immersed in room temperature water. To create a burn sepsis model, burned mice were subjected to intraperitoneal inoculation of Pseudomonas aeruginosa (2 × 105 CFU/ml, #27853, American Type Culture Collection, Manassas, VA, USA) at 1 day post burn as previously described [18]. Mice in Zn-MT-2 treated group were intraperitoneal injected with 0.5 mg/kg Zn-MT-2 (#M9542, Sigma-Aldrich, St. Louis, MO) twice a day, starting 1 day post burn immediately after the burn sepsis model was created, till 3 days post burn. Mice in Zn treated group received 35 μg/kg Zn (Because Zn-MT-2 contains 5–8 % Zn) as the same administration manner as Zn-MT-2 treated group. Animals were euthanized at 5 days post burn to assess the proinflammatory cytokines production and neutrophil infiltration in organs.

### Myeloperoxidase (MPO) assay

MPO activities in the liver and lung were measured as previously described [19]. Tissue samples were homogenized and centrifuged. 20 ml of the supernatant was incubated with 200 μL of substrate buffer. The optical density changes were read at 460 nm over a 2 min period by a microplate reader. MPO activities were obtained using a standard curve of purified MPO in the kit. Data are presented as units of activity per gram of tissue (U/g).

### Enzyme-linked immunosorbent assay (ELISA)

Interleukin (IL)-1β, IL-6, tumor necrosis factors (TNF)-α, and macrophage chemoattractant protein (MCP)-1 levels in serum were detected by commercially available ELISA kits (R&D Systems, Minneapolis, MN). All procedures were performed according to the manufacturer’s instruction book.

### Cell culture

After 6 days in culture, Raw 264.7 cells (ATCC, Manassas, VA) were stimulated with 10 % serum extracted from burn sepsis mice for 4 h. Cells were either co-cultured with Zn-MT-2 (2 μM, #M9542, Sigma-Aldrich, St. Louis, MO), equivalent concentrations of zinc sulfate, or Zn-MT-2 plus Akt inhibitor LY294002 (5 μM, #L9908, Sigma-Aldrich, St. Louis, MO). The mRNA expression of IL-1β and IL-6 were then detected by quantitative real-time polymerase chain reaction (PCR). Protein levels of Akt and phosphorylated Akt (pAkt) in cell lysates were determined by western blot.

### Quantitative real-time PCR

Total RNA was extracted using the RNeasy 96 Kit (QIAGEN, Hamburg, Germany). The cDNA was synthesized using a Superscript II reverse transcriptase (Invitrogen, Carlsbad, CA) and were amplified using the SYBR green PCR master mix (Applied Biosystems, Foster city, CA). Fluorescence was monitored and analyzed in an ABI 7500 system (Applied Biosystems, Foster city, CA). β-actin was used to normalize the data. All samples were analyzed in duplicate. The cycle threshold (Ct) values were obtained and the fold changes of gene expression were calculated by the 2^-ΔΔCt^ method [20]. The sequences of the primers for IL-1 β were Sense 5’- CTTCAGGCAGGCAGTATC-3’ and Antisense 5’-CAGCAGGTTATCATCATCATC-3’. The sequences of the primers for IL-6 were Sense 5’- CGGAGAGGAGACTTCACA -3’ and Antisense 5’- CTGTTAGGAGAGCATTGGAA-3’. The sequences of the primers for intercellular adhesion molecule (ICAM)-1 and vascular cell adhesion molecule (VCAM)-1 were Sense 5’-GGCTGGCATTGTTCTCTA-3’, Antisense 5’- TCCTCAGTCACCTCTACC-3’ and Sense 5’-GCGAGTCACCATTGTTCT-3’, Antisense 5’- GCCACTGAATTGAATCTCTG-3’, respectively. The sequences of the primers for β-actin were Sense 5’-GTCAGAAGGACTCCTATGTG-3’ and Antisense 5’- ACGCAGCTCATTGTAGAAG-3’.

### Western blot analysis

Protein levels in cell lysates were determined by bicinchoninic acid protein assay kit (Pierce Biotechnology, Rockford, IL). Protein was separated and transferred to a polyvinylidene difluoride membrane. The membranes were blocked and then incubated with anti-Akt (1:1000, # 9272, Cell Signaling Technology, Boston, MA) and anti-Phospho-Akt (Ser473) (1:1000, # 4060, Cell Signaling Technology). The membranes were then incubated with secondary antibody (1:2000; Invitrogen, Carlsbad, CA) for 1 h at room temperature. Immunoreactive bands were detected using an enhanced chemiluminescence reagent (Pierce Biotechnology) and exposed onto Kodak film (Eastman Kodak, Rochester, NY). Glyceraldehyde-3-phosphate dehydrogenase was used as a control. Band densities were quantified using Image J software (National Institutes of Health, Bethesda, MD).

### Statistical analysis

Data were expressed as means ± standard deviations (SD). Statistical analysis was performed by using Student's t test or one-way analysis of variance followed by Tukey’s test. A *p* value of less than 0.05 was considered statistically significant. GraphPad prism software V.6.01 for Windows (GraphPad Software, La Jolla, CA) was used for analyses.

## Results

### Effect of MT on inflammatory cytokines production

**Fig. 1:**
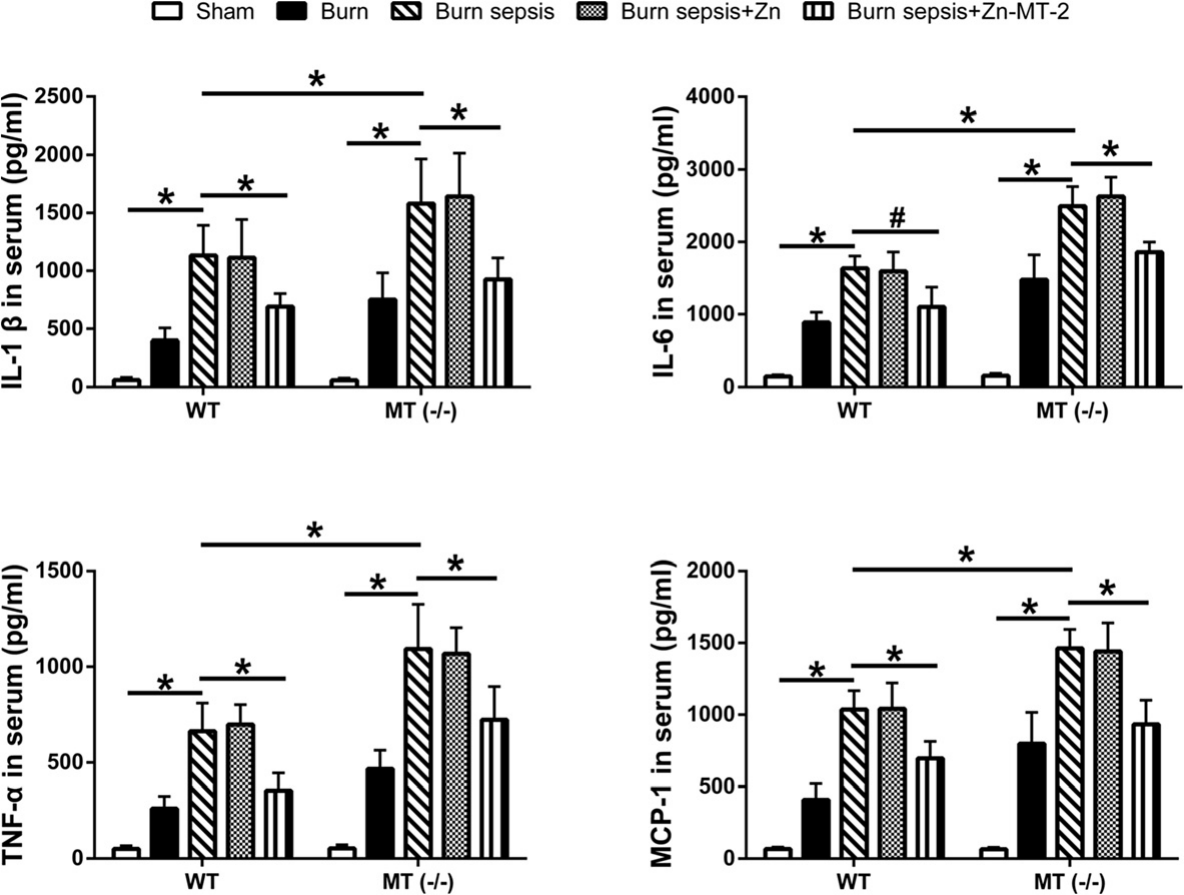
IL-1β, IL-6, TNF-α, and MCP-1 levels in serum of WT and MT (−/−) mice were detected by ELISA. Data were presented as means ± SD, *n* = 10 mice/group, *p < 0.01, #*p* < 0.05

Burn sepsis induced profoundly elevated levels of IL-1 β, IL-6, TNF-α, and MCP-1 in serum. The increase of these inflammatory cytokines were more prominent in MT (−/−) mice than in WT mice. Exogenous administration of Zn-MT-2 significantly inhibited these cytokines production after burn sepsis. But administration of Zn alone had no significant effect on the increase of these inflammatory cytokine levels (Fig. 1).

### Effect of MT on neutrophil infiltration in liver and lung

**Fig. 2:**
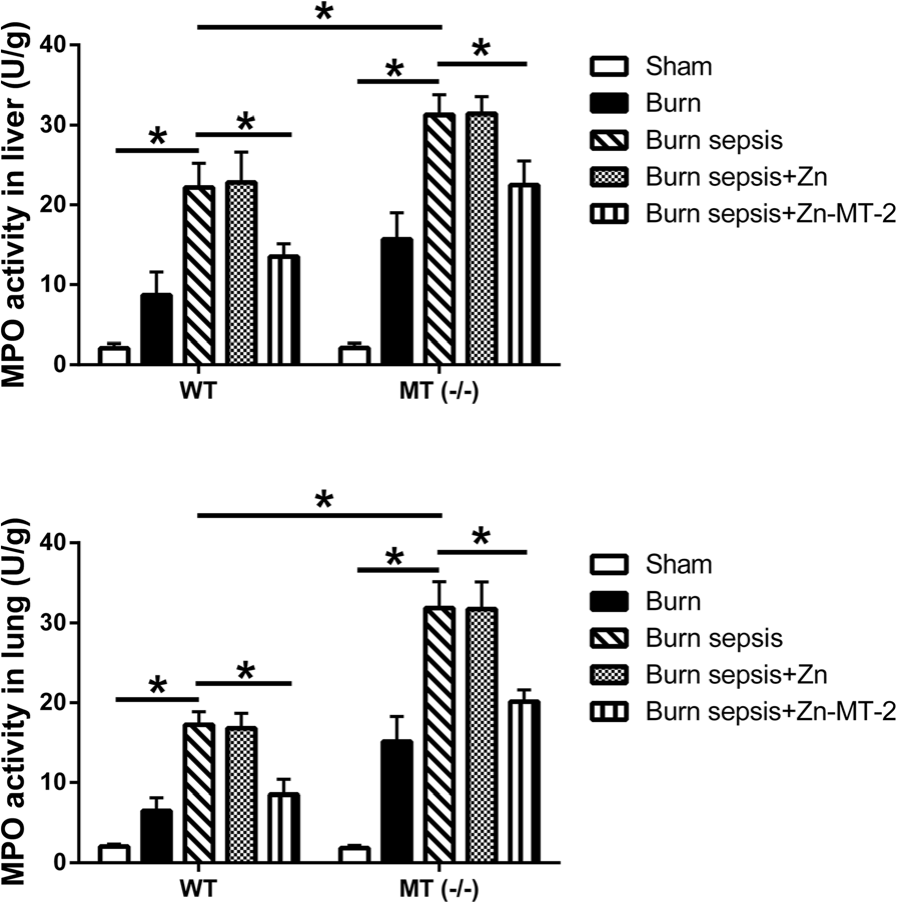
MPO activities in liver and lung of WT and MT (−/−) mice were detected by spectrophotometry. Data were presented as means ± SD, *n* = 10 mice/group, *p < 0.01

The MPO assay was performed to evaluate the neutrophil infiltration in liver and lung, the two organs that were among the mostly suffered from inflammatory damages after burn sepsis. Burn sepsis induced remarkably increased MPO activities in liver and lung. The increase of MPO activities were more prominent in MT (−/−) mice than in WT mice. Exogenous administration of Zn-MT-2 significantly decreased the MPO activities after burn sepsis. But administration of Zn alone had no significant effect on the increase of MPO activities (Fig. 2).

### Effect of MT on adhesive molecule expressions in liver and lung

**Fig. 3:**
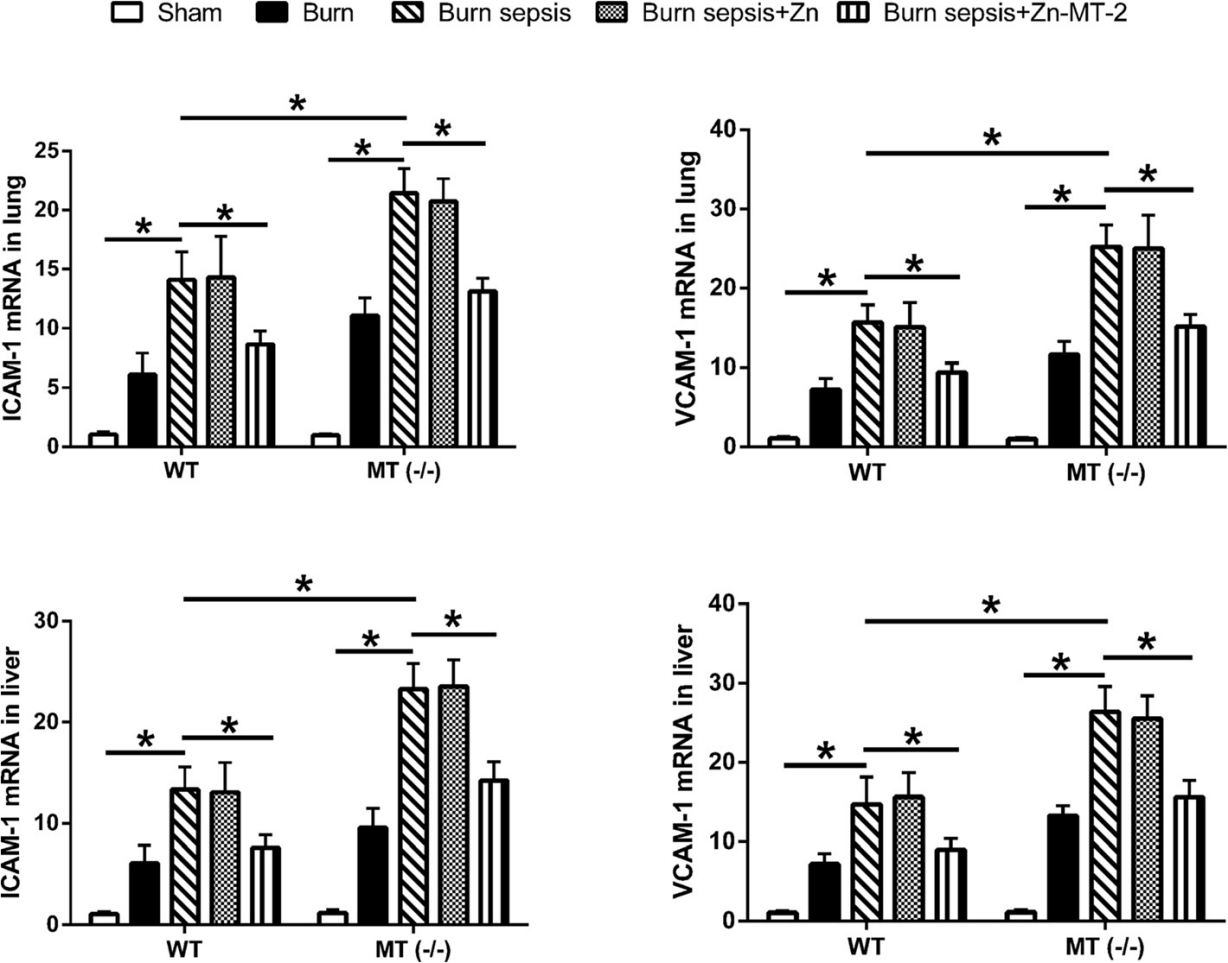
The mRNA expression of ICAM-1 and VCAM-1 in liver and lung of WT and MT (−/−) mice were detected by quantitative real-time PCR. Data were calculated using the 2^-ΔΔCt^ method, where Ct is cycle threshold. *n* = 10 mice/group, *p < 0.01

The mRNA expression of ICAM-1 and VCAM-1 in liver and lung were remarkably elevated after burn sepsis (Fig. 3). These increase of adhesive molecule expressions were more prominent in MT (−/−) mice than in WT mice. Exogenous administration of Zn-MT-2 significantly decreased the adhesive molecule expressions after burn sepsis. But administration of Zn alone had no significant effect on the increase of adhesive molecule expressions.

### The effect of MT was at least partly mediated by Akt signaling

**Fig. 4:**
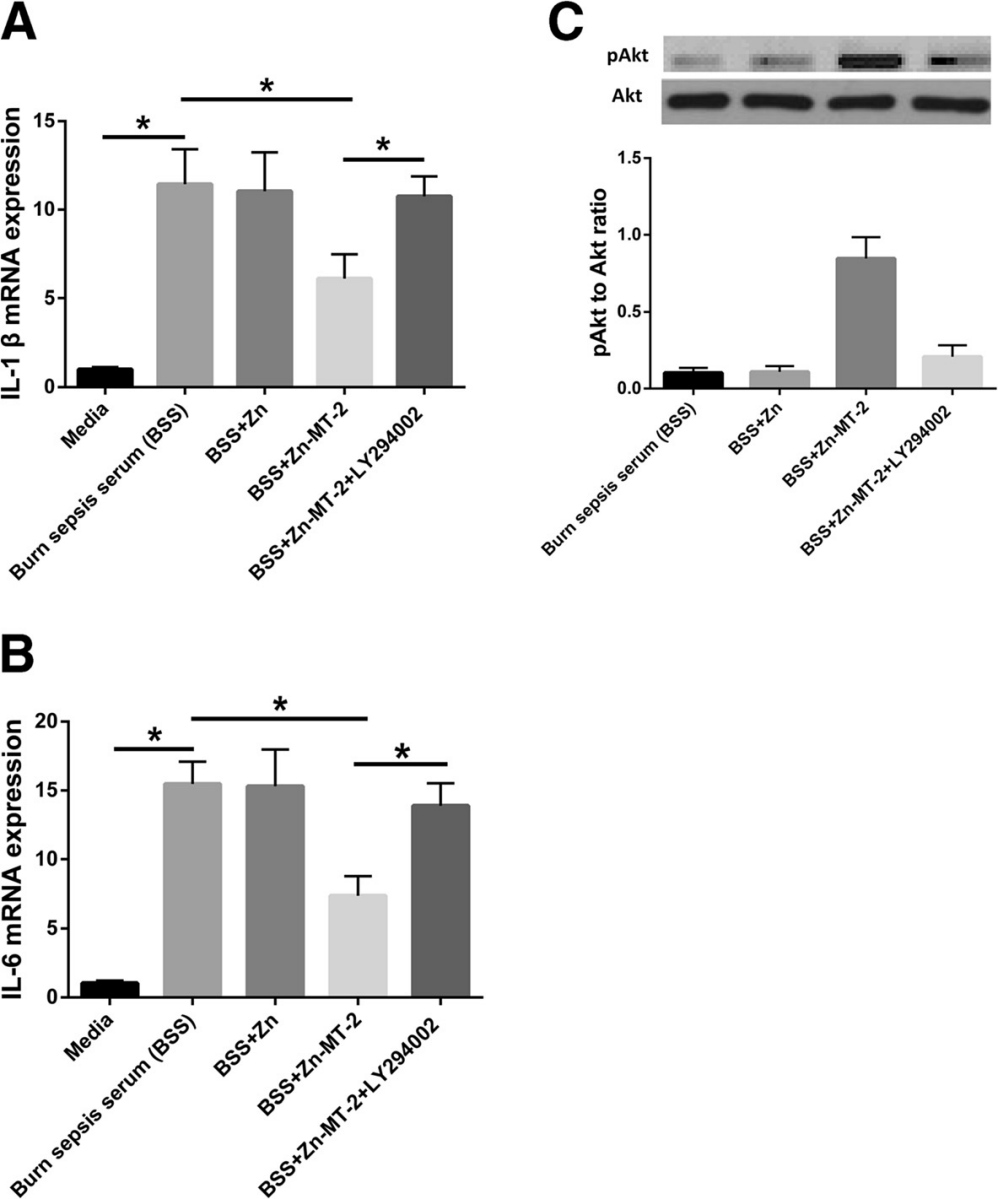
IL-1β (**a**) and IL-6 (**b**) mRNA expression in cell lysates were detected by quantitative real-time PCR. Data were calculated using the 2^-ΔΔCt^ method, where Ct is cycle threshold. **c** The protein levels of pAkt and Akt were determined by western blot. Data were presented as means ± SD. Results are representative of at least three independent experiments. *p < 0.01

The IL-1 β (Fig. 4a) and IL-6 (Fig. 4b) mRNA expression in Raw 264.7 cells were highly elevated after stimulation by burn sepsis serum. Zn-MT-2 upregulated the pAkt to Akt ratio and attenuated the increase of IL-1 β and IL-6 mRNA expression, whereas Zn alone had none of these effects. Moreover, the effects of Zn-MT-2 were abolished in the presence of LY294002.

## Discussion

Despite advances have been made in burn prevention, therapy, and rehabilitation, burn sepsis remains a major problem seriously affecting mortality and morbidity. The inflammatory response after burn is necessary for wound healing and host defense. Modest inflammatory response is important for the host to eliminate pathogens and promote wound repair [21]. However, major burns often evoke a systemic inflammatory response syndrome, which lead to reduced resistance to infection, development of sepsis and damage to multiple organs [22]. Our results in this paper have provided a novel potential therapeutic choice for the prevention of burn sepsis.

MTs have been found playing important roles in many physiologic and pathophysiologic situations ever since their discovery about fifty years ago [11, 23, 24]. Among these various effects of MTs, the role of MTs in inflammation and sepsis is gradually uncovered. MTs can be induced by various inflammatory stimuli and endotoxin challenge [25]. Reciprocally, MTs can regulate inflammatory response and attenuate endotoxemia [26]. MTs have been reported to have a beneficial effect in cardiac dysfunction in sepsis [14], neuroinflammation [27], intestinal inflammation [28], etc. However, the role of MTs in burn sepsis remains unclear.

In the present study, we investigated the role of MTs in burn sepsis using a mouse model. The results showed that MT (−/−) mice demonstrated a more prominent increase of inflammatory cytokine levels and more severe organ damage than WT mice after the sepsis challenge. These responses were inhibited by Exogenous MT-2 treatment to both MT (−/−) and WT mice, indicating a protective role of MT in burn sepsis.

In mechanistic study, MT-2 upregulated the pAkt to Akt ratio and abrogated the increase of IL-1 β and IL-6 mRNA expression from macrophages that stimulated with burn sepsis serum. The effects of MT-2 were abolished by inhibition of Akt phosphorylation level with LY294002. MT has been reported to prevent cardiac endoplasmic reticulum stress and cell death via activation of Akt signaling pathway. It seemed in this study that, the effect of MT in this mouse burn sepsis model is at least partly through activation of the Akt signaling pathway.

In conclusion, our results in this study provided evidence that MT-2 played a protective role against inflammatory response and organ damage in this mouse burn sepsis model, at least partly through activation of the Akt signaling pathway.
